# Health Literacy, Diabetes Prevention, and Self-Management

**DOI:** 10.1155/2017/1298315

**Published:** 2017-01-10

**Authors:** Joanne Protheroe, Gill Rowlands, Bernadette Bartlam, Diane Levin-Zamir

**Affiliations:** ^1^Keele University, Staffordshire, UK; ^2^Aarhus University, Aarhus, Denmark; ^3^Institute for Health and Society, Newcastle University, Newcastle upon Tyne, UK; ^4^University of Haifa, Haifa, Israel

## 1. Introduction

In the last few decades, focus on how to achieve and support maintenance of optimal glycemic control has become a well-established research area. It is well known and accepted that the degree of blood glucose control, especially in diabetes mellitus type 2, is linked with the risk of developing complications such as heart disease, stroke, renal failure, and blindness [1, 2]. Patients' knowledge about diabetes mellitus, their attitudes towards self-management, and self-management skills, together with lifestyle choices, are central to achieving and maintaining glycemic control, both in the short term and long term. The World Health Organization defines health literacy as “the personal characteristics and social resources needed for individuals and communities to access, understand, appraise and use information and services to make decisions about health” [3]. Health literacy is known to be associated with health outcomes, including chronic disease and diabetes [4]. Among people with type 2 diabetes, inadequate health literacy is independently associated with worse glycemic control, higher rates of retinopathy, and lower self-rated health [5, 6]. Inadequate health literacy may contribute to the disproportionate burden of diabetes-related problems among disadvantaged populations [6, 7]. A variety of measures have been developed to assess health literacy depending upon context, as reflected in the articles in this special issue.

Needing to understand this association in more depth and to explore potential interventions to improve diabetes health outcomes and quality of life was the basis for dedicating a special edition of this journal to such issues. The intention is to contribute to an evidence base that better informs diabetes treatment and prevention planning for patients, clinicians, and health policy decision-makers

The main themes highlighted in this issue are health literacy and lifestyle in relation to diabetes, including studies exploring behavior change and motivation; mechanisms, moderators, and mediators of change; interventions to promote healthy lifestyles with respect to diabetes; self-care and self-management including health promotion aspects of diabetes care; health literacy; the role of family, peer support, and other care-givers, social networks, and distributed health literacy; and health systems navigation and management. The 12 studies included reflect a global reach, with research from six countries across four continents.

*(a) Feasibility and Outcome Measures of Self-Management Interventions*. In their study from Spain, G. Moreno and colleagues describe their feasibility study conducted in a range of primary care clinics and show that the implementation of their diabetes self-management programme was feasible. However, it was shown that while self-efficacy, blood pressure, physical activity, and some dietary habits improved, glycemic control was not achieved, perhaps due to the short-term duration of the intervention in the context of a feasibility study. In another study, J. F. Graumlich and colleagues report on their randomized control trial testing the effectiveness of a medication planning tool implemented via an electronic medical record to investigate the improvement of people's medication knowledge, adherence, and glycemic control as compared to usual care. They found that people with diabetes type 2 who used the tool had greater knowledge of medication indication; however, there was no improvement in adherence nor in glycemic control. Yet it is reported that the tool supported patient-provider collaboration in the clinic and the authors hypothesize that results may be improved by extending the tool to home and community settings. In two linked papers reporting a feasibility randomized control trial into the use of Lay Health Trainers (LHTs) to support self-management in a population with low health literacy, J. Protheroe and colleagues show that the intervention was associated with improved mental health, and illness perception, in addition to better self-management skills and QALY profile at 7-month follow-up, while B. Bartlam and colleagues report on the mixed methods process study evaluation of the trial. They note that the intervention proved feasible and offered important insights for a follow-up randomized control trial, as well as further training to support the LHTs skills for counselling older people with diabetes. These studies further emphasize the difficulties with, and the importance of, recruiting participants with low health literacy into trials exploring the development of new interventions to improve self-management. Failure to include participants with low health literacy into such trials may in fact inadvertently worsen the disproportionate burden of diabetes-related problems among disadvantaged populations [6]. 

*(b) Culturally Sensitive and Ethical Aspects of Diabetes Risk and Self-Management Interventions among Special Populations*. Cultural considerations and appropriateness in the management of type 2 diabetes have been shown to be critical in offering appropriate care, particularly due to the cultural influences on lifestyle and self-management [8]. Several articles in this special issue focus on cultural aspects of diabetes management. E. Wilkinson et al. offer a literature review that examines the topic of older people with diabetes from ethnic minorities in the UK. They highlight emerging themes from the literature including cultural competency (health care providers understanding and taking account of the importance of social and cultural influences of patient health beliefs and behaviors) and comorbidity, with ramifications for “culturally intelligent” and individualized care. N. Patel et al. describe the pilot testing of a cross-cultural tool, adapting a self-assessment risk tool to the Indian Gujarati language in order to achieve an equivalent score, using a multicomponent translation model. In addition, C. T. Ing et al. report on a study in which Native Hawaiians and other Pacific Islanders who had received a culturally tailored, community based self-management intervention program (Partners-in-Care, PIC) were then randomized to social support groups or control to see if social support improved maintenance of the benefits of the intervention over time. While both the control and social support groups maintained their initial improvements over 6 months, the authors explore the further potential benefits of social support. 

*(c) Age and Gender Considerations*. The importance of individual background characteristics such as age and gender has also attracted the attention of researchers with regard to health literacy and diabetes self-management, as seen in two articles in this special issue. D. Goeman et al. have applied the Optimizing Health Literacy Access (Ophelia) approach for codesigning interventions based on tool for assessing health literacy specific needs of people/organizations. As reported in this issue, the method was successfully applied among older people with diabetes, through a home care nursing service in Australia. Gender issues regarding diabetes self-management have also been researched and are presented in this special issue. S. T. Hendriks et al. used the Patient Activation Model (PAM) to investigate gender differentiation and patient activation. Their findings showed no gender difference after adjusting for cofounders with regard to patient activation, while certain self-management factors in fact were related to gender. 

*(d) Health Literacy, Behavioral and Mental Health Risk Factors for Diabetes*. The behavioral risk factors for diabetes and the significance of lifestyle changes on the etiology of diabetes and on glycemic control are also critical issues for research [9–12]. Y.-T. Sung and colleagues explore the significance of smoking and smoking cessation on the incidence of diabetes. The findings showed that even up to 2 years subsequent to smoking cessation, ex-smokers still had a higher risk of diabetes. K. Friis et al. noted that, among people with diabetes in a Danish sample, lifestyle behaviors, namely, nutrition and physical activity were positively associated with health literacy, after controlling for confounders. Yet no such association was found between alcohol and tobacco consumption and health literacy. In the final article of this thematic area, D. Maneze et al. examined the influence of depression and health literacy on diabetes self-management and found that, among a sample in Australia, depressed mood predicted low health literacy and lower self-management. They concluded with a recommendation to screen for depression while helping also to support self-management among people with low literacy.

In summary, the articles in this special edition highlight important issues and emerging research in health literacy and in self-management in diabetes. This issue includes a particular focus on measurement and the cultural and ethical issues of self-management in populations at risk of poor outcomes, with examples from around the world. The importance of including these at risk populations in future trials of interventions, especially those designed to support self-management in diabetes, cannot be overemphasized. Further research is needed to ensure that the benefits for patients, communities, and societies can achieved through enhanced self-management strategies, and hence reduced risks of morbidity and mortality can be realized by all groups in societies, in diverse cultural settings.
